# A Panel of CpG Methylation Sites Distinguishes Human Embryonic Stem Cells and Induced Pluripotent Stem Cells

**DOI:** 10.1016/j.stemcr.2013.11.003

**Published:** 2013-12-26

**Authors:** Kevin Huang, Yin Shen, Zhigang Xue, Marina Bibikova, Craig April, Zhenshan Liu, Linzhao Cheng, Andras Nagy, Matteo Pellegrini, Jian-Bing Fan, Guoping Fan

**Affiliations:** 1 Department of Human Genetics, Broad Stem Cell Research Center, David Geffen School of Medicine, University of California, Los Angeles, 695 Charles Young Drive, Los Angeles, CA 90095, USA; 2 Illumina, Inc., 9885 Towne Centre Drive, San Diego, CA 92121, USA; 3 Translational Stem Cell Center, Tongji Hospital and Department of Regenerative Medicine, Tongji University School of Medicine, Shanghai 200092, China; 4 Department of Molecular, Cell and Developmental Biology, University of California, Los Angeles, Los Angeles, CA 90095, USA; 5 Stem Cell Program in Institute for Cell Engineering and Division of Hematology, Johns Hopkins University, Baltimore, MD 21205, USA; 6 Samuel Lunenfeld Research Institute, Mount Sinai Hospital, 25 Orde Street, 5-1015-3 Toronto, Ontario, Canada M5T 3H7

## Abstract

Whether human induced pluripotent stem cells (hiPSCs) are epigenetically identical to human embryonic stem cells (hESCs) has been debated in the stem cell field. In this study, we analyzed DNA methylation patterns in a large number of hiPSCs (n = 114) and hESCs (n = 155), and identified a panel of 82 CpG methylation sites that can distinguish hiPSCs from hESCs with high accuracy. We show that 12 out of the 82 CpG sites were subject to hypermethylation in part by DNMT3B. Notably, DNMT3B contributes directly to aberrant hypermethylation and silencing of the signature gene, *TCERG1L*. Overall, we conclude that DNMT3B is involved in a wave of de novo methylation during reprogramming, a portion of which contributes to the unique hiPSC methylation signature. These 82 CpG methylation sites may be useful as biomarkers to distinguish between hiPSCs and hESCs.

## Highlights

• Eighty-two DNA methylation sites distinguish hiPSCs and hESCs with high accuracy
• Twelve aberrantly hypermethylated signature CG sites are due in part to DNMT3B
• DNMT3B also contributes to previously reported aberrant hypermethylation in hiPSCs

## Teaser

Huang et al. identify a panel of 82 CpG methylation sites that can robustly distinguish hiPSCs from hESCs with high accuracy. Part of the signature consists of aberrantly hypermethylated CpG sites, which are partially abrogated in the absence of DNMT3B. In addition, DNMT3B-mutant hiPSCs do not exhibit other aberrant hypermethylation signatures as previously reported.

## Introduction

DNA cytosine methylation is a major epigenetic factor that contributes to regulating important biological processes such as genomic imprinting, X inactivation, and gene regulation. DNA methylation is established and maintained by a family of DNA methyltransferases (DNMTs), including DNMT1, DNMT3A, and DNMT3B, and deficiency in any Dnmt enzyme leads to embryonic death in mice. Additionally, aberrant DNA methylation is associated with human diseases such as cancer, immunodeficiency, and neurological disorders (Feng and Fan, 2009). Collectively, these observations indicate that DNA methylation plays critical roles in mammalian development.

DNA methylation is also important for both stem cell differentiation and cellular reprogramming. In differentiation, dynamic DNA methylation changes are critical for lineage specification as a wave of de novo methylation takes place to silence pluripotency genes and establish tissue-specific methylation patterns (Hawkins et al., 2010; Lister et al., 2009; Stadler et al., 2011). During reprogramming, DNA methylation contributes to an epigenetic barrier. Inhibiting the activities of DNMTs with 5-aza-cytidine (AzaC) or knocking down DNMT1 promotes partially reprogrammed cells into a fully reprogrammed state (Mikkelsen et al., 2008). Meanwhile, a wave of de novo methylation also occurs during reprogramming whereby tissue-specific genes and partially methylated domains (PMDs) become hypermethylated (Doi et al., 2009; Lister et al., 2009, 2011).

Induced pluripotent stem cells (iPSCs) have the characteristics of human embryonic stem cells (hESCs), and many studies have investigated the similarities between iPSCs and hESCs, including genome stabilities, transcriptome (Chin et al., 2009; Guenther et al., 2010; Newman and Cooper, 2010; Wang et al., 2011) and histone modifications (Guenther et al., 2010), and DNA methylation (Bock et al., 2011; Doi et al., 2009; Kim et al., 2010; Lister et al., 2011; Ohi et al., 2011; Ruiz et al., 2012). These studies revealed both similarities and differences in the properties of iPSCs and hESCs. DNA methylation in iPSCs has been reported to acquire irregular methylation patterns while retaining some memory of somatic cells during the reprogramming process, thus exhibiting a methylation profile unique to iPSCs (Bock et al., 2011; Doi et al., 2009; Kim et al., 2010; Lister et al., 2011; Ohi et al., 2011). However, because these previous studies differed in the quantitation techniques, genome coverage, and sample sizes employed, it remains contentious whether iPSCs possess a methylation signature that can be used to distinguish iPSCs from hESCs.

To address this issue, we systemically compared the methylation profiles of a large number of human iPSCs (hiPSCs) and hESCs from multiple labs to revisit the question of whether hiPSCs have a unique CpG methylation signature. We identified a panel of 82 CpGs that can distinguish hiPSCs from hESCs with high accuracy. Remarkably, 12 signature CpG sites tended to be hypermethylated compared with both somatic cells and hESCs, suggesting that these methylation signatures are not a form of residual somatic epigenetic memory. Hypermethylation of these 12 sites is partially disrupted in DNMT3B-deficient hiPSCs, consistent with the function of DNMT3B for de novo methylation during reprogramming. Together, our results demonstrate a robust hiPSC molecular signature that is partially a consequence of DNMT3B-mediated de novo methylation during reprogramming.

## Results

### A Unique DNA Methylation Signature Distinguishes hiPSCs from hESCs and Somatic Cells

**Figure 1:**
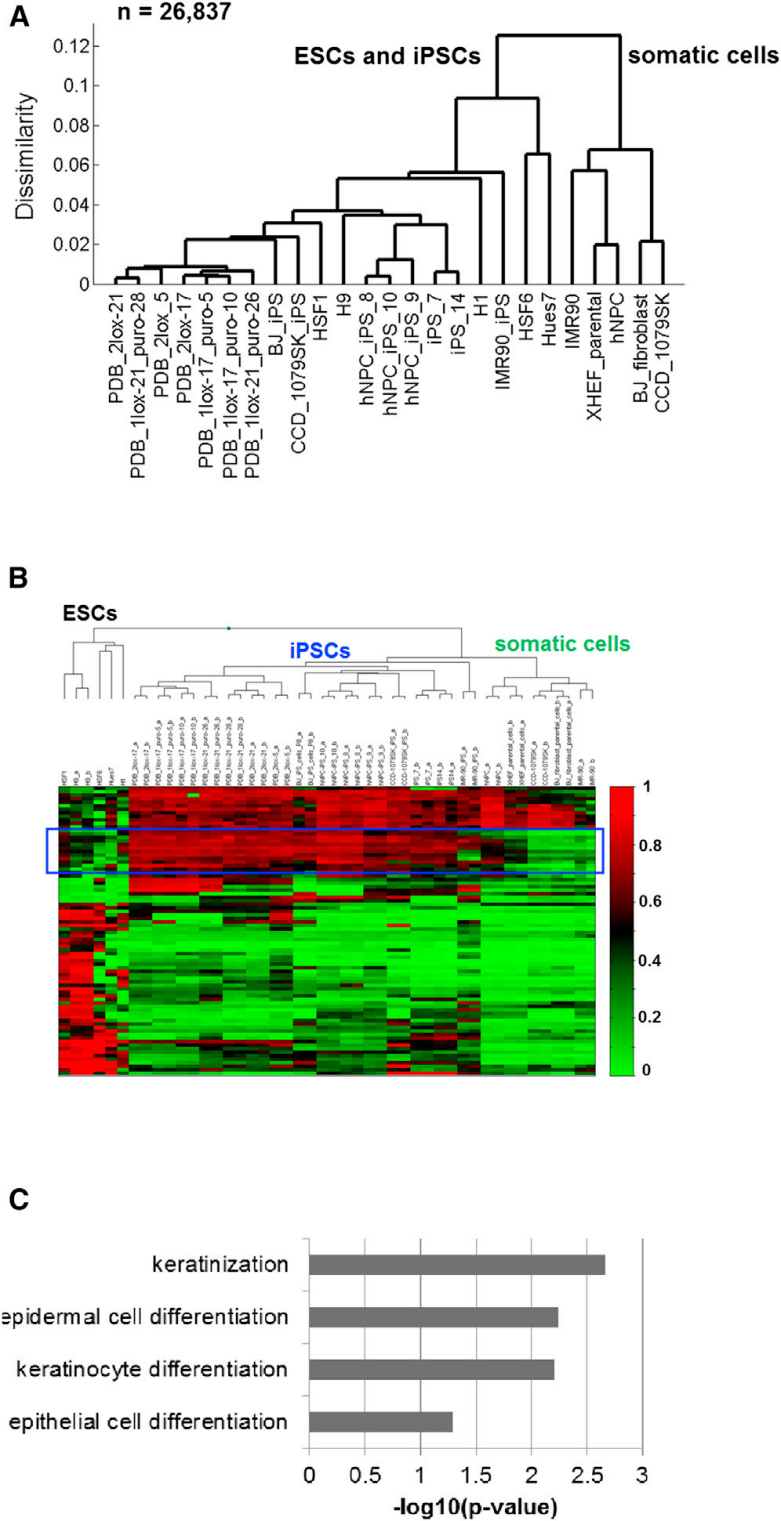
A Unique Signature Distinguishes hiPSCs, hESCs, and Somatic Cells (A) Clustering analysis of methylation profiling in 26,837 CpG sites in hiPSCs, somatic cells, and hESCs assayed by the Illumina Infinium platform. (B) Heatmap representation of the 82 signature CpGs in 15 hiPSCs, five hESCs, and five somatic cells. The blue box highlights CpGs that underwent de novo methylation in hiPSCs. (C) GO analysis of the signature genes (n = 66). The p values shown were adjusted using the Benjamini-Hochberg method. See also Figure S1 and Table S1.

In previous reports, investigators have debated whether hiPSCs exhibit a unique CpG methylation profile due to either residual somatic cell memory or aberrant methylation in select domains when compared with hESCs. In this study, we investigated this topic by analyzing CpG methylation in a large number of pluripotent cell samples (n = 269) from multiple labs, thus increasing the power of the statistical analyses. Using Illumina Infinium BeadChip assays, we first examined the methylation profiles of 25 cell lines, including five hESCs, five parental somatic cells, and 15 lines of hiPSCs covering hiPSCs generated by both vector-containing and vector-free methods. Globally, hierarchical clustering analysis demonstrated that hiPSCs are highly similar to hESCs, but distinctively different from somatic cells (Figure 1A). To identify differential methylation between hiPSCs and hESCs, we used a statistically stringent cutoff from Illumina’s custom model (see Experimental Procedures) and required an absolute methylation difference (delta-beta) of 0.3. We found that the methylation profiles from 82 CpG sites in 66 genes can effectively group hiPSCs separately from either hESCs or parental somatic cells (Figure 1B; Table S1 available online). Gene Ontology (GO) analysis revealed that the signature genes were associated with epidermal cell differentiation and keratinization (Figure 1C). Interestingly, comparisons of the hiPSC methylation signature among hESCs, hiPSCs, and somatic cells revealed that the hiPSCs’ methylation pattern often resembled that of somatic cells, except at 12 CpG sites that appeared to be uniquely hypermethylated compared with both hESCs and somatic cells (Figure 1B). This result suggested that the hiPSC methylation signature consists of both residual somatic memory and specific CpG sites that are subject to de novo methylation.

Ultimately, a robust signature should be able to accurately discriminate between hiPSCs and hESCs in independent data sets regardless of the laboratory of origin or the quantitation method used. Although hierarchal clustering is one way to visualize how multiple samples are grouped, we turned to more robust and quantitative classification methods. To provide an unbiased estimate of predictive accuracy for cell type, we used a leave-one-out analysis in which the support vector machine (SVM) learning model was fit on all but one sample and its prediction was related to the truly observed cell type of the left-out sample (see Experimental Procedures). Using other DNA methylation data available in the public domain, we consistently found that our signature CpGs could significantly improve accuracy over randomly selected CGs for identifying hiPSCs and hESCs. Although randomly selected CGs tended to have a relatively high accuracy, this appeared to be related to the level of hESC or hiPSC skew in the data set (data not shown). Nevertheless, in general, we observed correct classification of >95% of the samples with a false discovery rate (FDR) of <0.05. The results of the external validations are summarized in Table 1.

We found that our panel of CG signature sites was able to accurately discriminate between hiPSCs and hESCs despite the varying sample sizes among different studies. For example, we analyzed an independently derived Infinium 27k BeadChip data set that profiled DNA methylation in 20 hiPSCs and 11 hESCs (Chou et al., 2011), and showed a classification accuracy of 96%. Another study using the same platform examined 42 and 115 normal hiPSCs and hESCs (Nazor et al., 2012). In this larger data set, we could still accurately distinguish ∼97% of the hiPSCs and hESCs. Together, these results indicated that the methylation signature is robust to sample size sampling error.

Remarkably, our DNA methylation signature was also able to accurately discriminate between hiPSCs and hESCs regardless of the quantitation platform or technique used. In a data set compiled using the Infinium 450k BeadChip system, we examined DNA methylation in 29 hiPSCs and 18 hESCs (Nazor et al., 2012). Interestingly, although only 70 of the 82 signature probes were shared between the two Illumina BeadChip platforms, these 70 probes were still able to discriminate hiPSCs from hESCs with 97% accuracy. Next, we tried to cross-reference our signature CpGs with publically available genome-wide bisulfite sequencing data sets. Although the reduced representation bisulfite sequencing (RRBS) method is a cost-effective way to sample ∼3 million CpGs in the human genome (Bock et al., 2011; Ziller et al., 2011), we found that RRBS coverage had low overlap with the 82 signature CG sites (∼35 loci [∼40%] were detected) and was not ideal for cross-referencing. We therefore turned to whole-genome shotgun bisulfite sequencing data sets and curated a total of five hiPSCs and three hESCs generated from three separate labs (Chodavarapu et al., 2010; Laurent et al., 2010; Lister et al., 2009, 2011). Strikingly, methylation quantitation of the 82 signature sites through bisulfite sequencing could also separate hiPSCs from hESCs with 95% accuracy. Altogether, we analyzed 114 hiPSCs and 155 hESCs collected from multiple labs, and these results indicated that the identified CpG signature in our study is robust and can be broadly used to make a distinction between hiPSCs and hESCs.

### Pairwise Comparisons of Promoter CpG Methylation between hiPSCs and Somatic Cells Reveal a Wave of De Novo Methylation during Reprogramming

**Figure 2:**
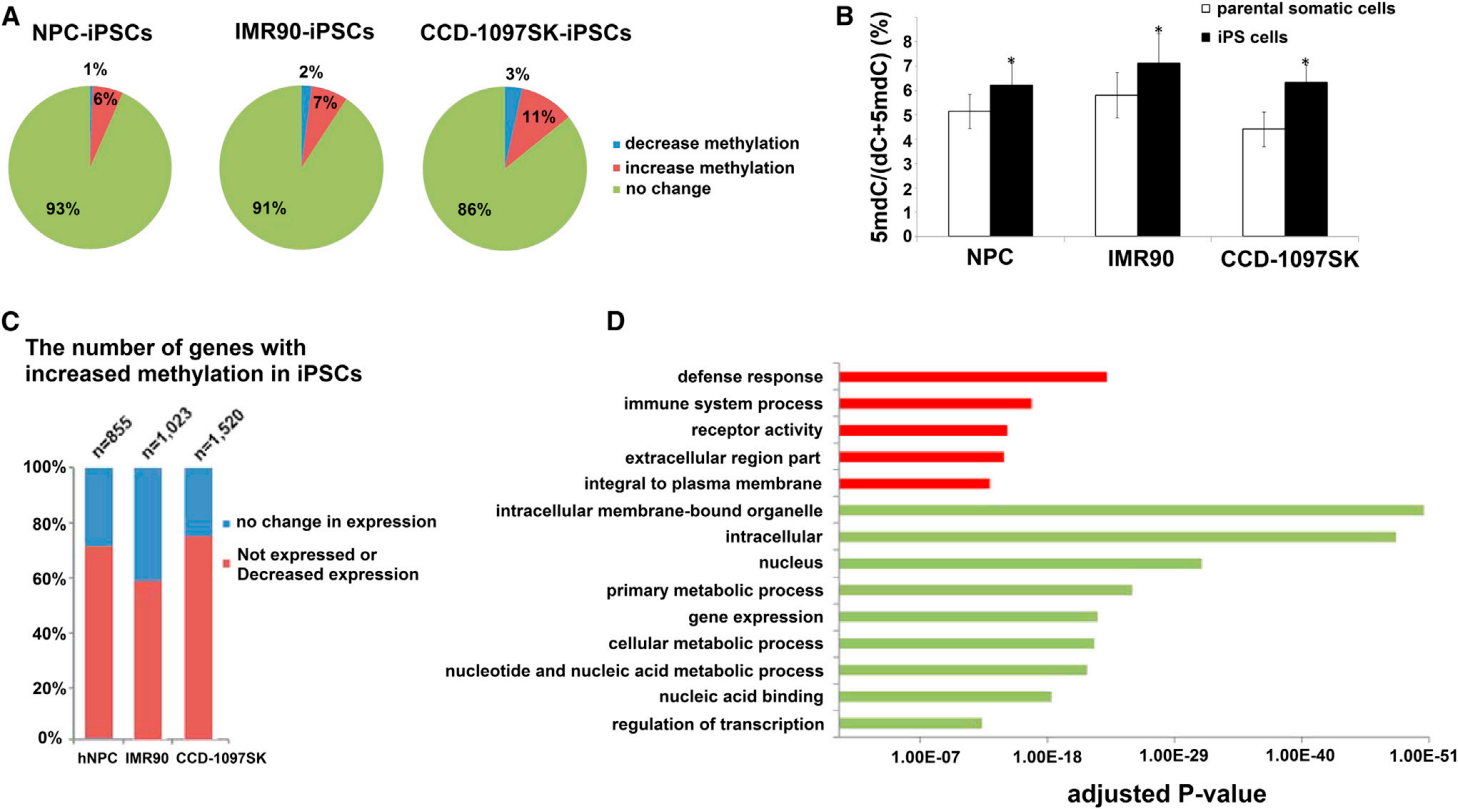
Pairwise Comparison between Parental Somatic Cells and hiPSCs Reveals Alterations of Promoter Methylation in Reprogramming and Correlation with Gene Expression (A) Global view of DNA methylation changes during the reprogramming of parental somatic cell lines to hiPSCs. Using a delta-beta > 0.3 (increase in methylation) or < −0.3 (decrease in methylation), we compiled the pie chart after comparing 26,837 CpG sites in 14,512 genes for each pair of somatic cells and hiPSCs. (B) Global methylcytosine levels as measured by HPLC-MS (n = 3 per sample). ^∗^p < 0.05 by Student’s t test. (C) Status of gene-expression changes between hiPSCs and somatic cells for the gene promoters showing increased methylation in hiPSCs. (D) GO analysis of genes with de novo methylation and decreased expression. The GO term is on the y axis and the p value indicating significance is on the x axis. The p values of GO terms that are overrepresented in the data set are colored in red; p values of underrepresented or depleted GO terms are colored in green (Benjamini-Hochberg adjusted p value < 0.05). See also Table S2.

Because different somatic cells could have different tissue- and cell-specific methylation patterns, we were interested in dissecting the precise methylation changes during the derivation of each iPSC line. We therefore performed a pairwise comparison in each CpG site between three pairs of hiPSCs and their parental somatic cells generated in our lab. We first identified genes that exhibited statistically significant changes in methylation pattern using a delta-beta value of ≥0.3 or a cutoff of ≤−0.3. Our data indicated that ∼7%–14% of gene promoters underwent methylation changes during direct reprogramming (Figure 2A), and 3.5- to 6-fold more gene promoters exhibited an increase in methylation than showed a decrease in methylation. This result is in line with previous observations describing a large number of hypermethylated promoters during reprogramming (Nishino et al., 2011), including hypermethylation at *MEG3*, *PEG3*, *ZIM2*, and other imprinted loci (Nazor et al., 2012). Indeed, high-performance liquid chromatography mass spectrometry (HPLC-MS) showed an overall increased level of DNA methylation in hiPSCs compared with parental somatic cells (Figure 2B). Notably, of the gene promoters with significant methylation changes during reprogramming (∼1,000), 11 (or 1%) were also associated with the hiPSC signature. Thus, a small portion of the hiPSC signature arises from global methylation changes during the reprogramming process, whereas the remaining portion of signature CpG sites are associated with somatic epigenetic memory.

By cross-referencing gene-expression profiles between hiPSCs and parental somatic cells, we found that ∼60%–75% of de novo methylation genes showed a significant reduction of gene expression (FDR < 10^−10^) or not expressed in hiPSCs (Figure 2C; Table S2). The increased methylation levels at promoter CpG sites in hiPSCs were confirmed by conventional bisulfite sequencing analysis (Figure S1). In total, 151 genes showed hypermethylation at promoter CpG sites and were also suppressed in all three pairwise comparisons. GO analysis indicated that these silenced genes were enriched for the genes required for specific functions such as immune system process and receptor activity (Figure 2D), consistent with a previous report (Nishino et al., 2011). These silenced genes were also depleted from genes involved in housekeeping functions such as intracellular membrane organelle, cellular metabolic process, and regulation of transcription (Figure 2D).

**Figure 3:**
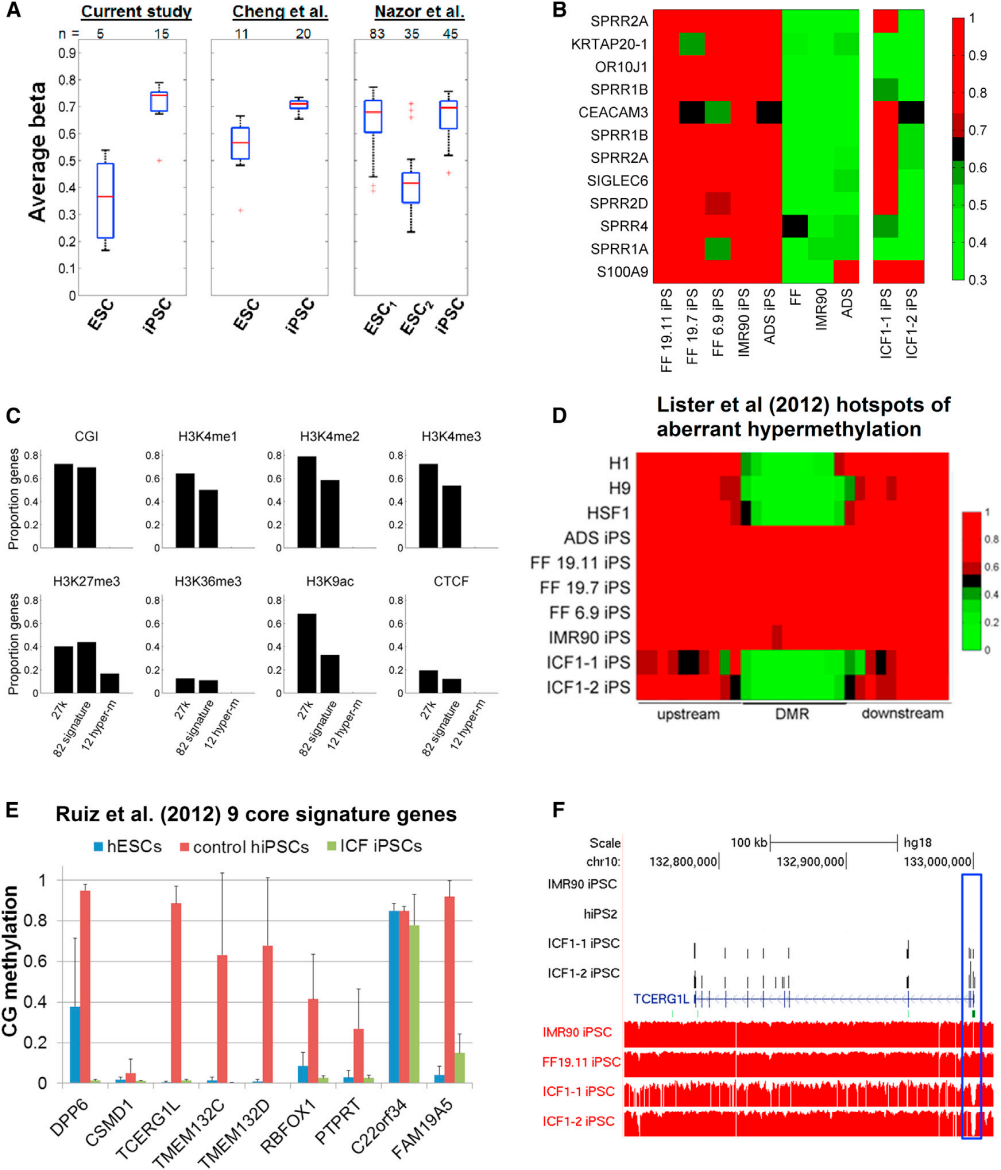
Wave of De Novo Methylation during Reprogramming by DNMT3B (A) Boxplot of the average methylation levels for the 12 sites that tend to be hypermethylated in hiPSCs. The + sign denotes outliers. ESC_1_ denotes hESCs from the CM, ESI, FES, SIVF, UC06, and MIZ series, and ESC_2_ denotes hESCs from the HES, WA, MEL, and MIV series. (B) Heatmap of the 12 CpGs through bisulfite sequencing. The legend represents raw methylation levels. (C) Bar graph of the percentage of sites that were positive for the labeled attributes. (D) Heatmap of CG methylation levels in previously identified domains (1–6 kb) of aberrant hypermethylation in iPSCs from Lister et al. (2011). (E) Bar graph of average CG methylation in the promoter (TSS ± 500 bp) of core iPSC signature genes from Ruiz et al. (2012). Error bars represent SD of the mean CG methylation in samples from WGBS data sets as described in Table 1. (F) Genome browser view of expression as measured by RNA sequencing (top four tracks) and CG methylation levels (red tracks) at the signature gene, *TCERG1L*. Note the selective hypomethylation at the proximal promoter associated with increased gene expression. See also Figure S2 and Table S3.

In addition to global hypermethylation, some of the methylation signature sites (n = 12) were also uniquely hypermethylated in hiPSCs, but hypomethylated in both somatic cells and a portion of hESCs (Figure 1B). Remarkably, these 12 sites were consistently hypermethylated in hiPSCs compared with hESCs in independent data sets (Figure 3A), confirming that these sites tended to show unidirectional differential methylation. However, on several occasions, we found heterogeneity in the methylation level at these 12 loci in hESCs (Figure S2). Interestingly, hESCs from the Nazor et al. (2012) data set revealed that cell lines from the CM, ESI, FES, SIVF, and UC06 series tended to be hypermethylated, whereas the HES, WA, and MEL series tended to be hypomethylated (Figure S2C). These differences did not appear to be laboratory dependent, since cell lines such as WA09 (also referred to as H9) cultured in four separate labs were consistently hypomethylated at these sites. Closer inspection of the Nazor et al. (2012) data sets revealed a mild inverse relationship between cell passage number and hypermethylation status (*r* = −0.42, p < 10^−7^; Figure S2D). Thus, prolonged culture appears to attenuate the hypermethylation at these 12 sites in a portion of hESCs. However, we have not analyzed methylation data of hiPSCs in extended culture (Table S3), so we cannot preclude the possibility that these 12 sites would show a similar epigenetic drift in long-term culture.

### Hypermethylation by DNMT3B Contributes to the Panel of Methylation Signatures

Because Dnmt3B is more dramatically upregulated in hiPSCs when compared with the levels of Dnmt3A and Dnmt1 (Stadtfeld et al., 2008), we hypothesized that DNMT3B may play a major role in de novo methylation in hiPSCs. To test this hypothesis, we generated hiPSCs from skin fibroblasts of patients with ICF (immunodeficiency, centromere instability, and facial anomalies) syndrome who carried double heterozygous point mutations in the catalytic domain of DNMT3B, and mapped the methylome for two ICF hiPSC lines at basepair resolution via whole-genome shotgun bisulfite sequencing. By cross-referencing other whole-genome bisulfite sequencing data sets, we confirmed that our 12 hypermethylation signature sites were hypomethylated in parental somatic cells, but hypermethylated in hiPSCs (Figure 3B). In the ICF hiPSCs, methylation levels at these 12 sites were generally reduced, but showed some variability (Figure 3B). Five of the 12 sites were consistently hypomethylated, suggesting that DNMT3B contributes to de novo methylation in at least some of these 12 CpG targets. We validated four out of five sites in additional ICF hiPSCs subclones using methylation-specific PCR (MSP) (Figure S2E). Remarkably, all 12 CpG sites were located in regions of low CG density (Figure 3C). In addition, by leveraging various histone peaks found in H1 ESCs, we found that these 12 CpG sites tended to also be devoid of histone marks (Figure 3C).

We next sought to determine how DNMT3B deficiency affects other hiPSC DNA methylation signatures reported in the literature. For example, kilobase hotspots for aberrant hypermethylation were previously identified in hiPSCs compared with hESCs (Lister et al., 2011). We found that all hotspot hypermethylated DMRs were hypomethylated in ICF hiPSCs and displayed a profile similar to that of hESCs (Figure 3D). Furthermore, eight out of nine previously identified core signature genes with aberrant promoter hypermethylation in hiPSCs (Ruiz et al., 2012) were found to be hypomethylated in ICF hiPSCs (Figure 3E). Notably, *TCERG1L* was consistently identified as an aberrantly hypermethylated gene in two previous studies and confirmed in our current study (Lister et al., 2011; Ruiz et al., 2012). Interestingly, gene-expression profiling of ICF hiPSCs and control hiPSCs showed that *TCERG1L* promoter hypermethylation is associated with gene repression (Figure 3F). Together, our data suggest that DNMT3B contributes to aberrant hypermethylation during cellular reprogramming.

## Discussion

Up to now, it was not clear whether hiPSCs have distinct transcriptomes and methylomes when compared with hESCs. Although one initial study reported the presence of iPSC-specific gene expression in a small number of iPSCs (Chin et al., 2009), several other studies argued that, at least on the individual gene-expression level, there are large variations among separate data sets (Guenther et al., 2010; Newman and Cooper, 2010). Recognizing the limitations for analyses based on individual genes, we previously utilized weighted gene coexpression network analysis (WGCNA) to identify functional modules that are distinct between iPSCs and ESCs (Wang et al., 2011). We further showed that one of these functional modules was inversely correlated with the level of DNA methylation in gene promoters, suggesting specific methylation changes in the hiPSCs. However, the module (n = 751 genes) had a small overlap (2 out of 66) with the signature genes identified in this study (*TCERG1L* and *TSPYL5*).

Because iPSCs exhibit a significant increase in genome-wide methylation when compared with parental somatic cells, we suspected that de novo methylation plays an important role in establishing a unique iPSC methylation signature. By comparing methylation patterns in mutant ICF-iPSCs, we indeed found some altered methylation signatures, suggesting that DNMT3B contributes to de novo methylation during reprogramming. In particular, we identified five signature CpGs (out of the 82 CpG signature sites) that undergo DNMT3B-mediated de novo methylation. This conclusion was also extended to hypermethylation signatures identified by others (Lister et al., 2011; Ruiz et al., 2012).

Our methylation signature is different from what was previously identified by either microarray or high-throughput sequencing analysis. Earlier studies suffered primarily from limited sample sizes due to the costly approach required to measure genome-wide DNA methylation levels on a comprehensive scale. Several previous studies using RRBS attempted to verify reported signatures in the literature and found a lack of reproducibility (Bock et al., 2011; Ziller et al., 2011), arguing instead for variations in iPSCs. Because RRBS covers ∼10% of human CpG sites and is biased toward regions of high CpG density, it is possible that the method could not fully detect the regions that were consistently different in iPSCs. A more recent study by Ruiz et al. (2012) using the bisulfite sequencing padlock probe (BSPP) system identified nine signature genes that distinguish hESCs from hiPSCs. On average, BSPP covers 500,000 CpGs in the human genome (∼1% of all human CpG sites); however, these sites have low overlap with the Infinium 27k array (∼25% shared sites within 100 bp). Moreover, when we compared our list of signature CG sites with other signatures in the literature, we found minimal overlap (Doi et al., 2009; Lister et al., 2011). Nevertheless, it is still unclear whether this low overlap is due to incompatible coverage or lack of sample size for robust delineation of an accurate signature. For example, Lister et al. (2011) initially identified hundreds of CG-DMRs in iPSCs, only a small fraction of which could be confirmed in multiple cell lines, suggesting that the number of sites is gradually reduced as the sample size becomes larger. By contrast, although we identified a methylation signature using 25 cell lines, we were able to validate these signatures in 249 other samples, demonstrating that our signature comprises a core set of CpG sites that can reliably distinguish iPSCs, hESCs, and somatic cells. Overall, we suggest that although a definitive signature whereby a given site is always differentially methylated between the two cell types may not exist, a panel of CpG sites representing loci that tend to be differentially methylated is sufficient to segregate iPSCs and hESCs. Thus, this panel of CpG methylation signatures in iPSCs may be useful as a molecular biomarker for classifying iPSCs in the future.

## Experimental Procedures

Briefly, hiPSCs were generated from IMR90, CCD-1097SK, BJ1, and NPC cells derived from 11-week-old fetal brain using retroviral expression of *OCT4*, *SOX2*, *KLF4*, and *c-MYC* or *OCT4*, *NANOG*, *KLF4*, and *LIN-28*. This study of hESCs and hiPSCs was approved by the UCLA Embryonic Stem Cell Research Oversight Committee. We used the HumanMethylation27 DNA Analysis BeadChip from Illumina to interrogate 26,837 CpG sites over 14,152 genes. Full experimental procedures and data analysis are available in the Supplemental Experimental Procedures.

## Figures and Tables

**Table 1:** Classification Accuracy of Signature Genes by SVM

Data sets	Platform	No. of Samples (hiPSC/hESC)	Accuracy		FDR (%)
			Random 82 (%)	Signature 82 (%)	
Huang et al., current study	Illumina 27k	20 (15/5)	81	100	4.55
Chou et al., 2011	Illumina 27k	31 (20/11)	69	96	0.01
Nazor et al., 2012	Illumina 27k	163 (45/118)	83	97	0.01
Nazor et al., 2012	Illumina 450k	47 (29/18)	82	97	0.31
Lister et al., 2009, 2011; Laurent et al., 2010; Chodavarapu et al., 2010	WGBS	8 (5/3)	68	94	7.10
	Total	269 (114/155)			

The FDR was determined by computing the accuracy of randomly selected 82 CG sites (n = 20,000) to generate a background (or null) distribution, and then finding the portion of the distribution that was greater than the observed accuracy of the signature sites.

## Accession Numbers

The microarray data have been deposited in the Gene Expression Omnibus under accession number GSE42043.

## Supplemental Information

Document S1. Supplemental Experimental Procedures and Figures S1 and S2

Table S1. Methylation Levels of 82 Signature Methylation Sites in hiPSCs, hESCs, and Somatic Cells, Related to Figure 1

Table S2. Gene-Expression Analysis of Genes with Promoter Methylation Changes during Reprogramming, Related to Figure 2

Table S3. Passage and Gender Information for Cell Lines Analyzed in the Current Study, Related to Figure 3
