# Supplementary material for: A Panel of CpG Methylation Sites Distinguishes Human Embryonic Stem Cells and Induced Pluripotent Stem Cells
Source: Stem Cell Reports. 2013 Dec 26;2(1):36–43. doi: 10.1016/j.stemcr.2013.11.003 (PMC3916755; doi:10.1016/j.stemcr.2013.11.003)
Supplement: Document S1. Supplemental Experimental Procedures and Figures S1 and S2 [file mmc1.pdf]

Stem Cell Reports, Volume *2*

Supplemental Information

A Panel of CpG Methylation Sites Distinguishes Human Embryonic Stem Cells and Induced Pluripotent Stem Cells

Kevin Huang, Yin Shen, Zhigang Xue, Marina Bibikova, Craig April, Zhenshan Liu, Linzhao Cheng, Andras Nagy, Matteo Pellegrini, Jian-Bing Fan, and Guoping Fan

Inventory of Supplementary information

The Supplemental Information contains extended Experimental Procedures and Supplementary Figures.

Figure S1 is a supplement figure to main Figure 1.

Figure S2 is a supplement figure to main Figure 3.

Table S1 is a supplement table to main Figure 1

Table S2 is a supplement table to main Figure 2

Table S3 is a supplement table to main Figure 3

Supplemental Experimental Procedures

Derivation and cultures of human iPSCs and hESCs

Human iPSCs were generated from IMR90 (ATCC), CCD-1097SK (ATCC), BJ1 (ATCC), and NPC cells derived from 11-week fetal brain (Shen et al., 2006) using retroviruses expressing OCT4, SOX2, KLF4, and c-MYC or OCT4, NANOG, KLF4 and LIN-28. Human ES cells were maintained in DME supplemented with 20% KSR, nonessential amino acids (Invitrogen), L-Glutamine (Mediatech), Penn/Strep, 2-mercaptoethanol with a feeder layer of MEFs as previously described (Shen *et al.*, 2006). For both gene expression and methylation analysis studies, the hESCs were passaged onto feeder free gelatin coated plates twice before harvesting RNA and DNA. RNA was isolated using Trizol (Invitrogen) while DNA was isolated using PureLink^{TM} genomic DNA purification kit (Invitrogen).

ICF1-1 and ICF1-2 iPSCs were derived from two different lines of ICF patient fibroblasts carrying non-conserved double heterozygous mutations in the catalytic domain. ICF1 iPSCs can form teratoma and differentiate into three germ-layer lineages, demonstrating the pluripotency of these iPSCs. The details of ICF iPSC characterization will be reported in a separate manuscript by **Huang et al. (in preparation)**.

DNA methylation profiling with Illumina Infinium assays

We have used the HumanMethylation27 DNA Analysis BeadChip from Illumina, Inc. (San Diego, CA) to interrogate 26,837 highly informative CpG sites over 14,152 genes. The human DNA sequence was based on the NCBI CCDS database (Genome Build 36) as described by the manufacturer. The experimental procedures of bisulfite conversion of genomic DNAs, hybridization of HumanMethylation27 BeadChips, and extraction of raw hybridization signals follow manufacturer's instruction. Data analysis was performed with the BeadStudio software

from Illumina, Inc. The assays were done in technical duplicates for each cell line, and exhibited very high correlations (average R^2 = 0.998).

Whole-genome gene expression analysis: humanht-12 beadchip and Agilent 44k

Gene expression microarrays were performed with Illumina Whole-Genome expression microarrays (HumanRef-8 v3.0 Expression BeadChip, Illumina, Inc. San Diego, CA) or Agilent 44K Whole Human genome arrays (G4112A; Agilent Technologies) using the suggested protocol. BeadStudio Software and Bioconductor package were used for data processing and analysis.

Clustering analysis of methylation data

The cluster analysis of methylation data was performed using Methylation Module v1.0 in BeadStudio (Illumina, Inc.) according to the manufactory's manual. Average signals of built-in negative control were used as the background value to normalize the methylation signals. Outliers are removed by using the median absolute deviation method. Methylation levels of individual loci in individual samples and sample groups were presented as beta values, which are estimated by calculating the ratio of intensities between methylated and unmethylated alleles. Differential methylation analysis algorithms and error models inherent in BeadStudio were used following the Illumina Custom Model. Briefly, the model assumes beta values are normally distributed, and p-values are defined as difference in mean and variance between beta values from two conditions (z-score). In general, differential methylation (or delta-beta) of >|0.3| are considered highly significant. Samples with differences of delta-beta value >|0.3| were therefore selected and subjected to the clustering analysis using cluster methods built in the BeadStudio software (Illumina, San Diego, CA).

Signature Validation and Data analysis

We performed quantile normalization for the Illumina 27k Infinium dataset from Chou et al. (2011) and Nazor et al. (2012). For the Illumina 450k Infinium dataset, we first performed peak correcting using the IMA package for R (Wang et al., 2012), followed by quantile normalization. For whole genome shotgun bisulfite data, we firstly mapped all raw reads to the hg18 genome using BS-Seeker (Chen et al., 2010). Next, we identified all CGs that fell within 5bp of each signature CpG site. If no CGs were found, we searched for CGs that were at most 100bp flanking the signature CpG site. For cell lines with no CGs found within this vicinity (i.e. missing data), we imputed the data point using nearest neighbor method only if a single data point was missing for a given gene. We did not perform any normalization on the bisulfite sequencing data, since these signals are inherently highly quantitative. For assessing promoter methylation (Figure 3d), we took the average methylation in the 500bp flanking the TSS.

Histone data is generated from ChIP-seq assays in H1 ESCs as part of the Epigenome Roadmap Project hosted by the UCSC genome browser. We defined positive sites as gene promoters (1kb around transcription start site [TSS]) that show statistically significant peak enrichment.

Support vector machine

Support vector machines are a type of classifier method based on hyperplanes in a high dimensional space in which samples can be separated by their distance from the planes. The R package 'e1071' was used to train the datasets and predict accuracy of classifying iPSCs or ESCs (linear kernel function). We used two-thirds data as training set, and the rest (33%) as test data. Leave-one-out cross-validation was performed using the 'cross' function (the parameter was set to equal the total number of samples) inherent in the R package. Accuracy was calculated as the portion of correctly classified samples in the SVM model. To generate the background (or null) distribution of a random signature, we calculated the accuracy of re-iteratively sampled 82

random sites (n= 20,000) and reported the mean of the distribution. False discovery rate (FDR) was determined by finding the portion of the distribution that was greater than the observed accuracy of the signature sites.

Bisulfite Conversion and Sequencing

Bisulfite conversion was performed as described (Shen et al. 2006). Briefly, we digested genomic DNA with BglII overnight. Digested DNAs were then incubated with a sodium bisulfite solution for 16 hours. Bisulfite treated DNA was then desalted and precipitated. We used 1/10 of precipitated DNA for each PCR. For PCR, we used nested primers to generate our products. PCR products were gel purified and used for Topo Cloning (Invitrogen).

Methylation-specific PCR

Genomic DNA was digested with MspJ1 enzyme (NEB) followed by PCR amplification using the following primers:

KRTAP-20F	TATGCCATCCAAAGGTGTCA
KRTAP-20R	TTCAATCACTTTCTGGTTGTCAG
OR10J1F	CTGAGCATGCTGTCCACTTC
OR10J1R	GCTGTGAGCAGGAAGCAGTT
SPRR1BF	CCTGCCAGGCACGAGTAT
SPRR1BR	GGGCTGTTTAATATATGAAGTGGA
SPRR4F	AGCTTGTCGCCTCTGGTAAG
SPRR4R	ACCCAAGGATGGAAGAGGAT
SPRR1AF	CCTCCCTTTCCCACCTATTC
SPRR1AR	GGGGACATCTGAGTCCTGTT

Gene ontology analysis

Gene Ontology analysis was performed using DAVID Bioinformatics Resource (Huang da et al., 2009) for Figure 1 and GOstat software (Beissbarth and Speed, 2004) for Figure 2. P-values in both cases were adjusted using the Benjamani-Hochberg method.

References

Beissbarth, T., and Speed, T.P. (2004). GOstat: find statistically overrepresented Gene Ontologies within a group of genes. Bioinformatics *20*, 1464-1465.

Chen, P.Y., Cokus, S.J., and Pellegrini, M. (2010). BS Seeker: precise mapping for bisulfite sequencing. BMC bioinformatics *11*, 203.

Huang da, W., Sherman, B.T., and Lempicki, R.A. (2009). Systematic and integrative analysis of large gene lists using DAVID bioinformatics resources. Nature protocols *4*, 44-57.

Shen, Y., Chow, J., Wang, Z., and Fan, G. (2006). Abnormal CpG island methylation occurs during in vitro differentiation of human embryonic stem cells. Hum Mol Genet *15*, 2623-2635.

Wang, D., Yan, L., Hu, Q., Sucheston, L.E., Higgins, M.J., Ambrosone, C.B., Johnson, C.S., Smiraglia, D.J., and Liu, S. (2012). IMA: an R package for high-throughput analysis of Illumina's 450K Infinium methylation data. Bioinformatics *28*, 729-730.

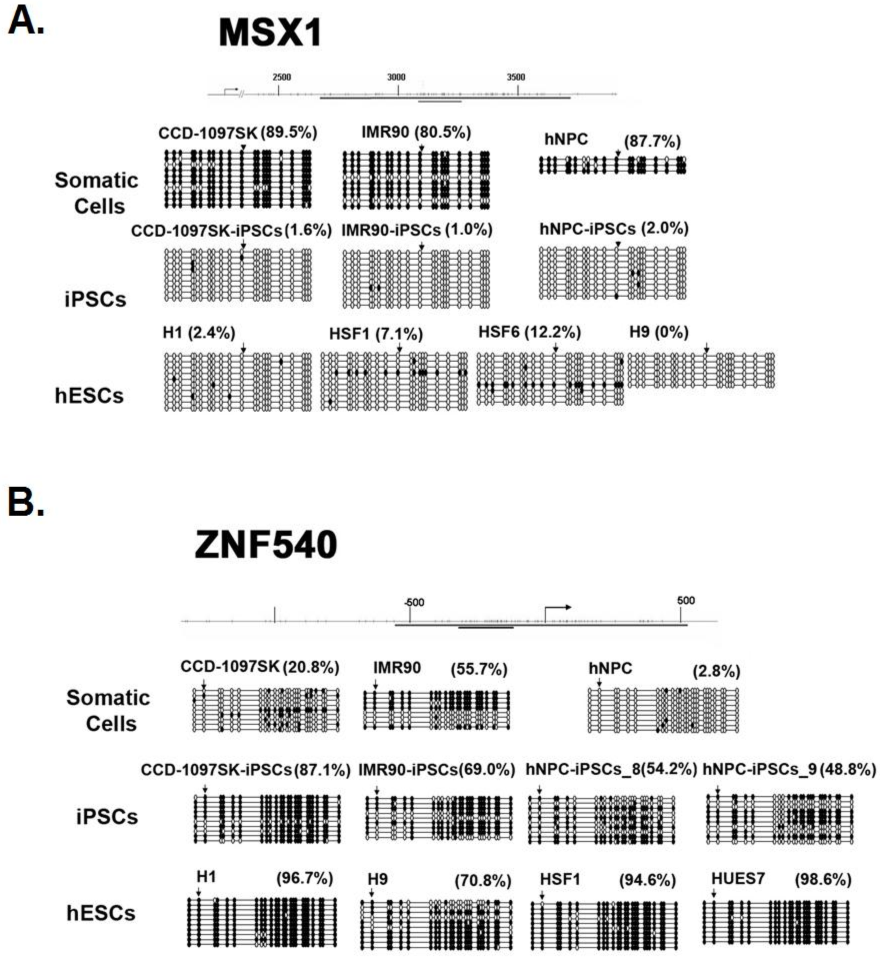

Supplemental Fig. S1. Conventional bisulfite sequencing validation. Bisulfite confirms dramatic changes in CG methylation in different cell types

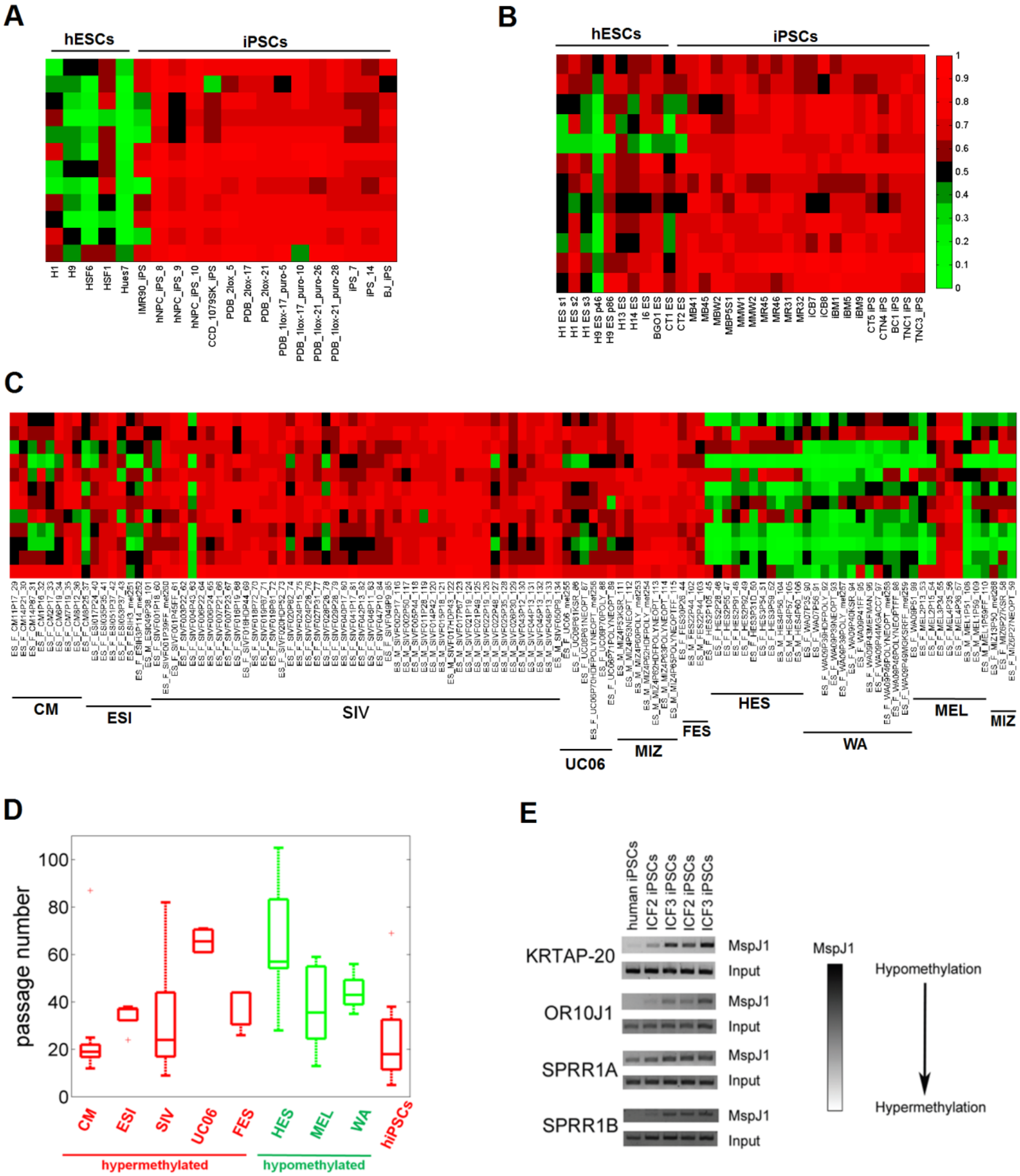

Supplemental Fig. S2. Hypermethylation of 12 signature sites in iPSCs. Heatmap representation of DNA methylation levels in A) Huang et al., B) Chou et al., and C) Nazor et al. For C), only ESCs are shown. ESCs appear to have variability at these 12 sites, and tend to cluster according to their cell series (e.g. CM, ESI, SIV, etc). D) boxplot showing the distribution of cell passage number for each cell series (e.g. CM, ESI, SIV, etc). Red and Green color indicates the methylation status of the 12 hypermethylated signature sites in hiPSCs. E) Methylation-specific PCR of 4 out of 5 CG hypermethylation sites. MspJ1 only cleaves modified cytosines and therefore only hypomethylated DNA would produce a PCR band. Primer sequences are listed in Experimental Procedures.
